# A multicenter feasibility randomized controlled trial using a virtual reality application of pain neuroscience education for adults with chronic low back pain

**DOI:** 10.1080/07853890.2024.2311846

**Published:** 2024-02-14

**Authors:** Ryan McConnell, Elizabeth Lane, Grace Webb, Dana LaPeze, Hailey Grillo, Julie Fritz

**Affiliations:** a Department of Physical Therapy, Belmont University, Nashville, TN, USA; b University of Utah, Salt Lake City, UT, USA; c Benchmark Physical Therapy, Chattanooga, TN, USA

**Keywords:** Musculoskeletal pain, psychological distress, physical therapists, virtual reality, chronic pain

## Abstract

**Background:**

Chronic low back pain (CLBP) is a highly prevalent condition among adults and is correlated to high levels of pain, high disability, and lower quality of life. Pain neuroscience education (PNE) helps to explain the pain experience and can affect psychosocial factors, such as fear of movement, anxiety, socioeconomic status, work life satisfaction, etc. More recently, virtual reality (VR) programs have emerged allowing for immersive PNE experiences.

**Objective:**

The purpose of this randomized clinical trial is to determine the feasibility of using a VR application for the delivery of immersive PNE (VR-PNE) and other activity training for patients with CLBP presenting to outpatient physical therapy (PT) clinics.

**Methods:**

A two-arm, parallel group, randomized controlled feasibility trial of patients was conducted at 12 outpatient PT clinics from March 9, 2022, through September 9, 2022. The intervention group received PT as usual and VR-PNE while the control group received PT as usual. Between group feasibility, acceptability outcomes and other patient-reported outcomes were assessed at six weeks.

**Results:**

A total of 595 individuals were evaluated for low back pain during the recruitment period. Seventy individuals were eligible and met definition for CLBP, 52 enrolled and 32 completed the trial. Participant adherence was 63.6% for VR-PNE and 63.2% for PT as usual. Participants found VR-PNE acceptable and reported satisfaction scores (0–100) of 87.37 ± 11.05 compared to 81.17 ± 23.72 in the PT as usual group. There were no significant differences between groups for the BBQ, BRS, FABQ-PA, FABQ-W, GROC, NPRS, NPQ, PCS, and PSEQ at 6 weeks.

**Conclusion:**

The results of the trial suggest that VR-PNE may be acceptable and feasible for patients with CLBP. Study procedures and PT delivery modifications should be considered for the next iteration of this study to improve follow-up assessment rates.

## Introduction

Chronic low back pain (CLBP) is a problem that has a large global and economic impact [1]. In the United States alone, reports indicate that CLBP affects approximately 28% of adults each year and 90% across their lifespan, with a financial burden of over $300 billion annually [1]. Low back pain lasting greater than 90 days is correlated to high levels of pain, high disability, and lower quality of life [2, 3, 4, 1, 5]. Successful outcomes for CLBP pain are highly influenced by multiple psychological factors, such as depression, low resilience, low pain self-efficacy, and anxiety [6, 7, 5].

Due to the complex nature of chronic pain, lack of consistent physical causes for persistent disability, and poor outcomes of traditional therapies, it is imperative that alternative approaches to treatment are developed. Pain neuroscience education (PNE) is an excellent adjunct to physical therapy (PT) to assist in addressing chronic pain [8] and is recommended in current clinical practice guidelines [9]. PNE is a multidimensional educational strategy that has been around for decades and helps patients to understand the pain experience as a multidimensional matrix [10, 11]. It has been shown to decrease kinesiophobia and pain catastrophizing [12]. More recently, combining PNE with exercise has demonstrated greater improvements in pain, disability, kinesiophobia and pain catastrophizing as compared to exercise alone for chronic musculoskeletal pain [13]. A variety of delivery methods have been utilized, including in-person, telehealth, articles/pamphlets, etc [14].

Virtual Reality (VR) is a developing technology that has been shown to effectively reduce chronic low back pain through therapeutic mechanisms including distraction, neuromodulation of body perception and graded exposure therapy [15, 16]. In addition, VR can be a great non-pharmacologic approach for managing chronic low back pain as it has been shown to improve patients’ pain intensity, mood, quality of life, and functional abilities [2]. VR-delivered PNE may provide an effective way for PNE to be administered, reduce burden [17] on clinicians, and make PNE more accessible to patients [18]. Additionally, because many clinicians feel unprepared or uncomfortable with the psychosocial aspects of chronic low back pain [19] a standardized education format could provide some structure to a complicated topic. PNE is traditionally delivered in conjunction with other treatments to provide a comprehensive treatment program [8]. More recently, the development of VR programs has emerged, allowing an immersive PNE experience [20]. A call for more research to determine the benefits of using VR for managing chronic pain conditions in inpatient and outpatient has been established [15, 21].

The purpose of this randomized clinical trial was to evaluate the feasibility of delivering PNE through VR for patients with chronic low back pain presenting to outpatient PT clinics.

## Materials and methods

### Trial design

The study was a two-arm, parallel group, randomized controlled feasibility trial of patients and was conducted in Tennessee. IRB exemption status was granted through University of Utah and Belmont University (IRB_00145358 and IRB_1200). It was determined that PT and PNE pose no more than minimal risk to participants and are commonly used. The trial was prospectively registered (clinicaltrials.gov NCT05285462).

### Modifications from registration

The original patient reported outcomes included the Working Alliance Inventory (WAI), which was not recorded due to unintentional omission during REDCap entry. Additionally, data were captured and reported for therapist demographic data, knowledge and attitudes about pain and VR-PNE system specific feedback to better understand the characteristics of therapist teams that had high engagement with this trial.

### Participants and randomization

Participants were recruited from 12 outpatient PT clinics in middle Tennessee presenting with a primary complaint of CLBP from March 9, 2022, through September 9, 2022. Inclusion criteria included age 18–75 and LBP greater than or equal to 12 weeks. Exclusion criteria included recent lumbar surgery, neurological condition or compromise, certain systemic diseases, or currently known to be pregnant. The inclusion and exclusion criteria are listed in Table 1.

**Table 1.** Inclusion and exclusion criterion.

Inclusion	Exclusion
18–75 years old Pain in low back >12 weeks	Systemic metabolic condition Neurological or muscular degenerative disorder Epilepsy Systemic infection Spinal surgery (<12 months) Spinal pathology such as stenosis or spondylolisthesis Spinal fracture Acute radiculopathy or compromised nerve root Pregnancy

Participants were randomized to an intervention group that included PT as usual augmented by virtual reality pain neuroscience education (VR-PNE) or a PT as usual through a block scheme in Research Electronic Data Capture (REDCap), a web-based, Health Insurance Portability and Accountability Act compliant platform (Vanderbilt University, Nashville, TN, version 11.0.3).

### Interventions

All participants received standard PT directed by physical therapists. Participants were treated in clinics with a frequency and duration left to the physical therapists’ discretion with no interference by the study team to maintain pragmatism.

**Figure 1.**
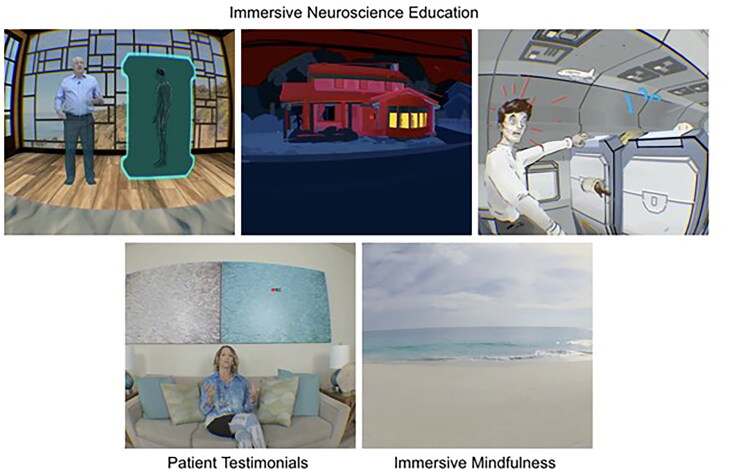
PNE 2.0 content modules – BehaVR proprietary content are reproduced with the permission of BehaVR.

**Figure 2.**
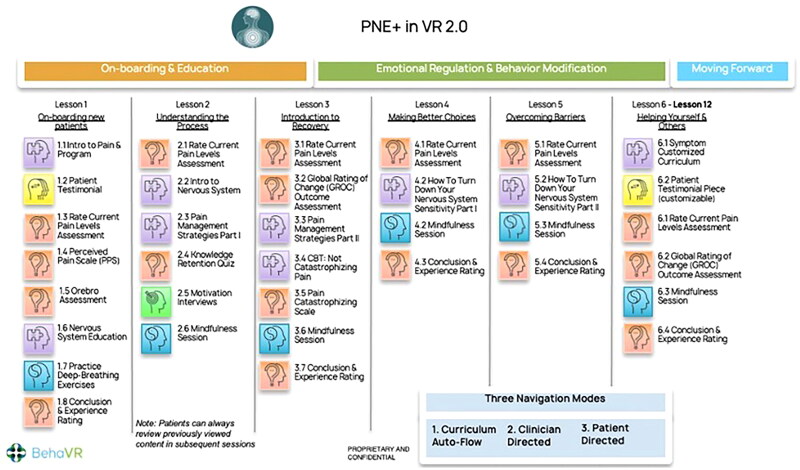
PNE 2.0 software schedule.

PNE 2.0 software (BehaVR Inc., Elizabethtown, KY, version 2.0) was delivered using a consumer grade PICO G2 4K VR head-mounted display (PICO Interactive, San Francisco, California, U.S.A.). PNE 2.0 is a 12-session VR-PNE program for chronic pain that uses both immersive real-world footage and interactive computer-generated imagery (CGI) to deliver visually and emotionally engaging education and relaxation training activities (Figure 1). VR-PNE combines traditional pain education modules, with customizable patient testimonials, and interactive emotional regulation practices such as breathing and guided mindfulness exercises in six different natural environments. The mean session time is approximately 21 min. Session content leverages VR technology by delivering PNE through artificial interactions with experts and by using engaging content such as patient testimonials, emotional regulation techniques (e.g. mindfulness and breathing exercises) and is detailed in Figure 2 and Appendix A1. Each clinic was provided two systems to utilize in the event of multiple patients requiring VR-PNE intervention or to address technical issues.

Participants randomized to the VR-PNE group received adjunctive PNE at the clinic during their routine PT visits. Similarly, VR-PNE session frequency varied per physical therapist discretion but could be included with each clinic visit. Prior to the second PT session, baseline outcomes were recorded. All participants received the same sequence of lessons and individual sessions were not tailored. At six weeks, participants had the following measures and tests repeated (Table 2). Participants were allowed to continue VR-PNE sessions beyond six sessions. Compliance in each group was assessed post-hoc by examining electronic medical record and VR-PNE software. Each participant’s total number of visits to the clinic were counted and compared to the VR-PNE record of the total number of VR-PNE sessions completed.

**Table 2.** Outcome measures.

Outcome measure	Description
**Oswestry Disability Index** [22]	Patient-completed questionnaire which gives a subjective percentage score of level of function (disability) in activities of daily living in those rehabilitating from low back pain. The ODI shows good internal consistency (α = 0.85). Discrimination of all the items is high to perfect (1.08–2.01).
**Numeric Pain Rating Scale (NPRS)** [23]	Ranges from 0 (no pain) to 10 (worst imaginable pain) as a measure of pain intensity. The NPRS has excellent test-retest reliability for LBP (0.61) and neck pain (0.76).
**Pain Self-Efficacy Questionnaire (PSEQ)** [24]	Quantifies an individual’s confidence in performing activities despite pain. The PSEQ contains 10 items ranked from 0 (“not at all confident”) to 7 (“completely confident”). The PSEQ has high validity when compared with measures of coping (*r* = 0.48) and pain beliefs (*r* = 0.74) and excellent test-retest reliability (0.73).
**Pain Catastrophizing Scale (PCS)** [25]	Quantifies the catastrophic thoughts regarding pain. Scores range from 0 to 52, with higher scores indicating higher levels of pain catastrophizing. The PCS was found to be highly reliable (Cronbach alpha = 0.75 to 0.86) and have good criterion-related, concurrent, and discriminant validity.
**Global Rating of Change (GROC)** [26]	Is used to gain the patient’s perceived progress of their condition since the beginning of PT. The GROC ranges from +7 (a very great deal better to 0 (about the same) to) −7(a great deal worse). The GROC is a valid measure of measuring a patient’s perceived change in quality of life.
**Neurophysiology of Pain Questionnaire (NPQ)** [27]	Quantifies the patient’s knowledge of physiology of pain.
**Fear Avoidance Beliefs Questionnaire (FABQ)** [28]	Assesses fear and avoidance beliefs related to physical activity (FABQ-PA) and work activity (FABQ-W). Is a 16-item questionnaire to rate their agreement with each statement on a seven point scale. The total FABQ has been shown to correlate with a measure of disability and fear avoidance.
**Brief Resilience Scale (BRS)** [29]	A measure of a person’s ability to bounce back or recover from stress.
**Back Beliefs Questionnaire (BBQ)** [30]	Measures patient’s attitudes and beliefs towards recovery and return-to-work; and expectations regarding the negative circumstances that could be created as a result of low back pain (LBP).

### Site recruitment

Initial interest in study was queried during the physical therapists’ regional operations meeting and a list of interested clinic directors provided contact information. One month later, ongoing interest in the study was assessed and the clinic directors who remained interested were included as final host locations. Therapists’ information was also collected, which included demographic data, including age, race/ethnicity; practice and educational details; confidence levels about PNE, VR-PNE and research procedures; and therapists’ knowledge and attitudes about pain.

### Staff training

Prior to the start of recruitment, all participating physical therapists were provided an in-person, three-hour training session by the lead investigator. The session included the following information on the study: study rationale with review of PNE efficacy, recruiting strategy and procedures. The training session did not include formal PNE instruction for any clinical staff. Product training included a demonstration of the web-platform, technical product features and a live practice session of the VR-PNE. Physical therapists were provided supporting documents that included a live presentation, links to screening forms, product support information, and standardized communication scripts for recruitment.

All office staff were provided with a one-hour digital training session that included study rationale and instructions for utilizing digital screening forms. A standardized study invitation communication script was provided with a frequently asked questions support document. A second one-hour training session was performed at the three-month mark to refresh office staff. All study staff and physical therapists received the primary investigators’ contact information for ongoing questions and/or concerns.

### Clinic recruitment motivational strategies

At the end of each month, the clinic that invited the most participants received 50-dollar gift cards for all staff and new goals for recruitment were established. Emails were sent out by the study team weekly with a scorecard for each clinic and included answers to frequently asked questions. Clinics with low recruiting efforts were individually called to discuss obstacles and barriers. Two regional meetings were held to review study procedures, answer questions and to brainstorm ideas to improve recruiting efforts.

### Participant recruitment and study follow-up

All patients with any duration of low back pain were initially invited by clinical staff to receive information from research staff regarding potential participation in the study. All invitations were recorded by clinical staff regardless of interest in the study or chronicity of back pain. An electronic receipt of this recording was emailed to research staff. Research staff called those interested to educate about the study, provide an opportunity to answer questions, and determine eligibility. If agreeable to participation, the consent form with baseline survey was emailed to the participant during the call. If the individual did not complete the study forms, the research staff called the next day as a reminder. This process was repeated for up to three reminder calls. Participants were deemed ineligible for enrollment in either group after completing two visits of PT.

Before visit two, after digital consent and eligibility were confirmed, baseline measures were captured directly through REDCap *via* a link emailed to participants. The following outcomes were recorded at baseline: (1) Demographic data, including age, sex, race/ethnicity, employment status and general medical and LBP history; (2) Oswestry Disability Index (ODI) [22], Numeric Pain Rating Scale (NPRS) [23], psychosocial covariate measures [Fear Avoidance Belief Questionnaire (FABQ) [28], Pain Catastrophizing Scale (PCS) [25], and Pain Self-Efficacy Scale (PSEQ) [24], Brief Resilience Scale (BRS) [29], Neurophysiology of Pain Questionnaire (NPQ) [27]^,^ and the Back Beliefs Questionnaire (BBQ) [30]. Descriptions of baseline and outcome tools can be found in Table 2.

Participants completed PT as they normally would with no interference from the study team. Visit attendance was recorded in the electronic medical record and documented by study staff. If applicable, the number of VR-PNE sessions completed was recorded by the VR-PNE headset. Follow-up assessments were collected at six weeks after enrollment. Links to the survey were sent out *via* automatic, emailed invitations through REDCap. Study staff contacted participants *via* phone call if study assessments were not filled out within 24 h. Up to three calls were made.

All project data were entered into REDCap to provide easy data manipulation with audit trails for reporting, monitoring and querying records, and an automated export mechanism to common statistical packages (SPSS, SAS, Stata).

### Sample size

Because the objectives of this study related to feasibility, a pre-determined recruitment period was used versus a power analysis. The recruitment period was set for six months to assess feasibility outcomes. We conducted a power analysis to determine the number of patients that would be required to detect a small effect size. A sample size of 278 patients would be needed for a t-test (between two independent groups) with alpha error of 0.10 to achieve an effect size of 0.30 on disability with 0.80 statistical power calculated by G*power (Heinrich-Heine-Universität Düsseldorf, Düsseldorf, Germany, version 3.1).

### Statistical methods and data analysis

SPSS (IBM, Armonk, NY, U.S.A., version 28) was used for statistical procedures. Baseline categorical variables were provided as frequency counts in tables. Continuous variables were summarized in tables with descriptive statistics. Summary tables report all patient reported outcomes mean differences, 95% confidence intervals and mean error estimates. P-values were reported for individual interpretation but should be reviewed with caution because the study was not adequately powered to detect between group differences.

### Primary aims: feasibility and acceptability outcomes

**Figure 3.**
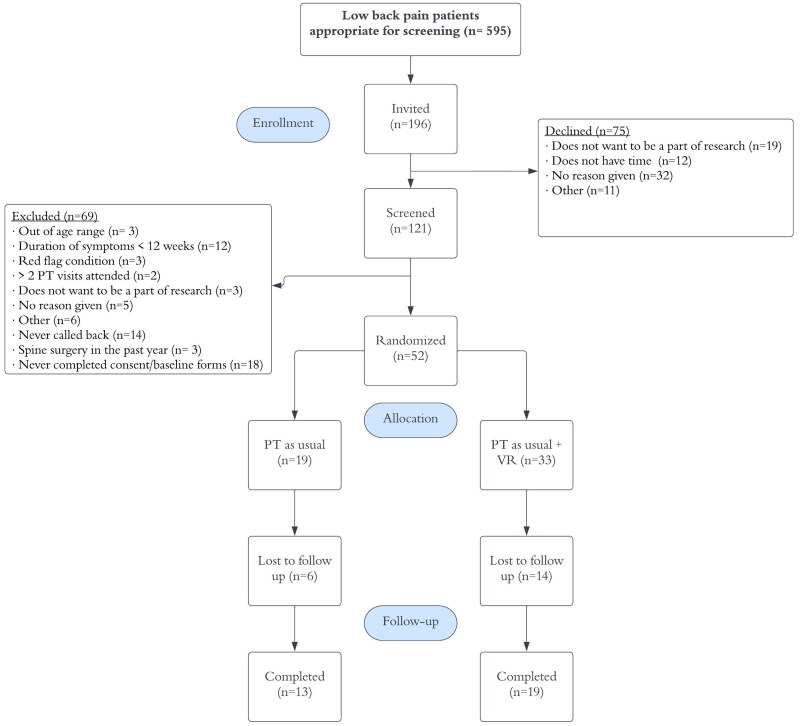
Flow of participants through the trial.

Recruitment rates were measured as percentages for each recruitment step. The *invitation rate* was calculated by dividing the number of individuals with low back pain that were invited to the study by clinical staff by the number of lumbar evaluations completed by participating sites over the six-month recruitment period. The s*creening rate* was calculated by dividing the number of participants who were screened by research staff/number of patients with low back pain that were invited to the study by clinical staff. *Eligibility rate* was calculated by dividing the number of patients who were eligible and sent initial surveys (included consent) by the number of participants who were screened by research staff. *Consent rate* was calculated by dividing the number of participants that were consented and randomized by the number of patients who were eligible and sent initial surveys (included consent). Reasons for invitation and screening refusal were collected as able and described in Figure 3.

*Research-procedure adherence* was measured as a rate of participants that completed follow-up assessments by group allocation. *PT visit attendance* was calculated by counts of PT visits attended in both groups. *VR-utilization* was measured as a percentage of participants in the VR-PNE group completing at least six VR-PNE sessions. *Acceptability* of treatment measures included patient satisfaction comparisons between the two groups. Participants were asked “How satisfied were you with receiving VR-PNE as part of your PT care” and “How satisfied were you with your PT care”. Other measures and descriptions can be found in Table 2.

### Secondary aim 1: to describe the between groups differences for outcome measures (BBQ, BRS, FABQ-PA, FABQ-W, GROC, NPRS, NPQ, PCS, and PSEQ at 6 weeks)

Baseline and follow up dependent variables had missing data. Missingness was assessed with Little’s Missing Completely at Random test (MCAR). A non-monotonic pattern of data missingness was handled using Markov Chain Monte Carlo multiple imputation method. The most appropriate statistical procedures (multivariate Kruskal-Wallis test) were unavailable due to the multiple imputation method. To describe between group differences, separate independent t-tests analyses were conducted for the BBQ, BRS, FABQ-PA, FABQ-W, GROC, NPRS, NPQ, PCS, and PSEQ. The NPRS was pooled as an average of the best, current, and worse and reported subsequently. Means and error measurements were reported for hypothesis generation.

## Results

### Participants and group allocation

Baseline characteristics are provided in Table 3. Inclusion and exclusion criteria are given in Table 1. Nineteen participants were allocated to PT as usual and 33 to VR-PNE. Participant allocation was unbalanced due to unequal clinic participation using 4-block randomization.

**Table 3.** Characteristics of the patient sample by treatment group.

Characteristic	All patients (*n* = 32)	VR-PNE (*n* = 19)	No VR-PNE (*n* = 13)
Age, years (SD)	46.2 (14.7)	48.2 (12.7)	43.3 (17.4)
Sex, (n, % male)	12 (37.5%)	8 (42.1%)	4 (30.8%)
BMI, kg/m^2^ (SD)	34.0 (10.7)	34.3 (12.3)	33.7 (8.5)
Marital status, (n, % married or live with significant other)	22 (68.8%)	14 (73.7%)	8 (61.6%)
Education, (n, % with college degree)	12 (37.5%)	7 (36.8%)	5 (38.5%)
Race/ethnicity (n, %)			
American Indian/Alaskan Native	1 (3.1%)	1 (5.3%)	0 (0%)
Black/African American	7 7 (21.9%)	3 (15.8%)	4 (30.8%)
White/Caucasian	23 (71.9%)	14 (73.7%)	9 (69.2%)
Other	1 (3.1%)	1 (5.3%)	0 (0%)
Hispanic or Latino	2 (6.3%)	2 (10.5%)	0 (0%)
Current smoker (n, %)	9 (28.1%)	6 (31.6%)	3 (23.1%)
History of depression (n, %)	12 (37.5%)	4 (21.0%)	8 (61.6%)
History of anxiety (n, %)	14 (47.8%)	6 (31.6%)	8 (61.6%)
Duration of back/neck pain, number of weeks (SD)	306.9 (372.6)	281.0 (349.1)	344.8 (416.3)
Receipt of prior treatment, N (%)			
No	11 (34.4%)	7 (36.8%)	4 (30.8%)
Yes	21 (65.6%)	12 (63.2%)	9 (69.2%)
Mean LBP, NPRS, 0–10	5.8 (2.1)	5.5 (1.9)	6.1 (2.4)
Oswestry Disability Index Score, 0–100 (SD)	35.0 (17.5)	34.7 (17.2)	36.9 (18.2)
Brief Resilience Scale, 6–36 (SD)	11.7 (1.7)	11.4 (2.0)	11.9 (1.1)
Pain Catastrophizing Scale 6-item, 0–24 (SD)	9.3 (5.3)	8.8 (5.2)	10.1 (5.4)
Total NPQ, % correct (SD)	44.8 (19.0)	41.7 (16.7)	49.4 (22.0)
FABQ physical activity, 0–24 (SD)	13.5 (5.8)	11.8 (6.0)	15.8 (5.0)
FABQ work, 0–42 (SD)	14.1 (12.6)	15.3 (13.1)	12.4 (12.1)
Pain Self-Efficacy Questionnaire 4-item, 4–24 (SD)	13.5 (7.2)	12.5 (8.1)	15.0 (5.6)
Back Belief Questionnaire, 9–45 (SD)	30.9 (8.1)	29.0 (8.4)	33.7 (7.2)

Missing data information: 2 participants missing BMI; 2 missing ethnicity; 1 missing smoking; 2 missing depression; 3 missing anxiety; 5 missing pain duration; 2 missing previous treatment; 1 missing ODI; 1 missing FABQ-PA; SD Standard Deviation; BMI Body Mass Index; LBP Low Back Pain; NPQ Neurophysiology of Pain Questionnaire; FABQ Fear Avoidance Belief Questionnaire.

### Primary aims: recruitment, invitation, eligibility, and consent rates

A total of 595 individuals were evaluated for low back pain during the six-month recruitment period. Of these individuals, 196 were invited to the study by clinical staff. Sites SBY, SMY, SH accounted for 54.5% of the invitations. Of the 196 invited to the study by clinical staff, 121 individuals (61.7%) were screened by research staff. Reasons for study refusal and exclusion are shown in Figure 3. Of the 121 individuals screened by research staff, 70 individuals (57.9%) were eligible and sent consent documents and baseline surveys. Of the 70 individuals who verbally agreed to participate, 52 individuals (74.3%) consented and were randomized.

### Study and PT adherence rates

Table 4 illustrates details of group adherence. Of those randomized 19 VR-PNE participants (57.6%) and 13 PT as usual (68.4%) returned follow up assessments. Fourteen participants in the VR-PNE group and six in PT as usual were lost to study follow up for unknown reasons. Participants attended an average of 9.97 visits (SE,1.03) in the VR-PNE group and 8.35 visits (SE,1.77) in the PT as usual group, respectively. Reasons for discontinuation of PT care are detailed in Table 5.

**Table 4.** PT adherence and study adherence.

Group	PT visits mean (SE)	% completing ≥ 6 PT or PT + VR-PNE sessions	VR-PNE sessions (SE)
VR-PNE (*n* = 33)	9.97 (1.03)	63.6	7.05 (0.73)
PT as usual (*n* = 19)	8.35 (1.77)	63.2	

* data for “did not return follow up” were captured post-hoc through VR headset and chart review.

**Table 5.** Reasons for discontinuation of PT care.

Rationale for discontinued care	
VR-PNE	PT as usual
“Doctor discharged”	“Didn’t have time to go”
“Heart pain”	“Insurance”
“I can do the same PT at home”	“Lost my vehicle due to not being able to work”
“I have another pelvic floor therapy I have to go to”	“Pain and copay; per visit and being disabled!”
“Other complications”	
“Stopped having pain”	

### VR utilization

Overall, 21 of 33 allocated to VR-PNE (63.6%) participated in at least six VR-PNE sessions regardless of follow-up assessment status. Of those who completed the study follow-up assessment, 73.7% completed at least 6 VR sessions. Fifty percent of those who did not complete the study follow up assessment still completed at least six VR sessions (Table 4).

Participant satisfaction between VR (mean, 87.37 SD: [11.05]) and PT as usual (mean, 81.17 SD: [23.72]) groups at six weeks is reported in Table 6. No adverse events were detected during the VR-PNE or PT sessions.

**Table 6.** Satisfaction and acceptability of VR.

Question	VR mean	Standard error	PT as usual mean	Standard error
How satisfied were you with VR-PNE as a part of your care?	75.89	4.69	NA	NA
How well do you feel that the VR-PNE education you received fit with the PT care (talked about similar messages, time management issues, logistical issues, added value/ was redundant	76.29	5.25	NA	NA
How satisfied were you with your PT care	87.37	2.74	81.17	6.76

### Secondary aim 1: describe differences between groups by outcome measures (BBQ, BRS, FABQ-PA, FABQ-W, GROC, NPRS, NPQ, PCS, and PSEQ at 6 weeks)

A Little’s MCAR test revealed random missingness (*p*=.30). Independent t-tests demonstrated no statistically significant difference between the BBQ (-3.31, CI: [-8.21, 2.15], *p*=.25), BRS (-1.05, CI: [-2.67, 0.58], *p*=.21), FABQ-PA (-2.36, CI: [-2.36, 1.89], *p*=.21), FABQ-W (0.05, CI: [-6.71, 6.81], *p*=.98), GROC (-0.78, CI: [-2.95, 1.39], *p*=.48), ODI (3.23, CI: [-5.31, 11.77], *p*=.46), NPRS (0.43, CI: [-1.09, 1.95], *p*=.58), NPQ (-5.07, CI: [-20.22, 10.07], *p*=.51) PCS (-2.11, CI: [-6.26, 2.04], *p*=.32), and PSEQ (-3.26, CI: [-8.56, 2.04], *p*=.23) at the six week follow-up in those who received PT versus PT and VR-PNE (Table 7).

**Table 7.** Independent samples t-test.

Variable	VR-PNE initial	VR-PNE follow up	PT as usual initial	PT as usual follow up	Mean difference between groups	Standard error difference	95% CI lower	95% CI upper	*p* value
BBQ	29.00	28.81	33.69	30.48	−3.31	2.64	−8.21	2.15	.25
BRS	11.47	12.49	11.92	11.89	−1.05	0.83	−2.67	0.58	.21
FABQ-PA	11.79	11.99	15.77	13.61	−2.36	1.89	−2.36	1.89	.21
FABQ-W	15.26	19.03	12.38	16.20	0.05	3.45	−6.71	6.81	.98
GROC	*	10.61	*	9.83	−0.78	1.11	−2.95	1.39	.48
NPQ	41.67	46.23	49.36	48.85	−5.07	7.71	−20.22	10.07	.51
NRPS	5.54	4.10	6.05	5.04	0.43	0.77	−1.09	1.95	.58
ODI	34.91	27.97	36.97	33.26	3.23	4.35	−5.31	11.77	.46
PCS	8.79	11.39	10.15	10.65	−2.11	2.12	−6.26	2.04	.32
PSEQ	12.53	16.88	15.00	16.10	−3.26	2.71	−8.56	2.04	.23

* No baseline value captured.

## Discussion

The present study describes the feasibility of using a randomized design to assess VR-PNE in addition to traditional PT for those with CLBP. Issues with research methods and intervention methods that affected our outcomes were identified.

In evaluating the research methods, the low rate of study outcome completion is a major concern. Given that many participants continued with PT and VR-PNE intervention but did not complete study outcomes, it is likely that our study processes were sub-optimal. Some explanations might include ease of accessing and completing study assessments, time commitment for completing study assessments, a lack of compensation for completing assessments, and lack of engagement by clinical staff. Study assessments were collected through REDCap, which emails a link to the participant. Some participants noted technical issues with broken links from REDCap emails. Study staff were notified in some of these cases, but the back-and-forth communication and problem solving caused many participants to give up on completing surveys. It is possible that many gave up after experiencing one broken link and did not notify the study team. Sending surveys *via* SMS text messaging may survey response rates [31,32]. Surveys completed over the telephone may have higher completion rates than email alone, and, if email is used, the completion rate is much higher when participants are called to notify the individual of the email [33].

Time required to complete assessments may also have played a role. REDCap does not consistently document the time for study assessments to be completed. In the cases where this information was available, the time ranged from 10 to 30 min for the baseline assessment. Short forms were used where possible to reduce burden, however some time may be saved by more parsimonious selection of outcomes. Lastly, the clinical staff was burdened by an acquisition that changed several policies during our recruitment period. This process cost the staff a considerable amount of time and mental energy that likely detracted from keeping participants engaged. To address the issues above, our study team will need to expand to include personnel for calling and remaining engaged with participants and secure funding to renumerate participants for their time of completing assessments.

Issues with usual PT care and the VR intervention were also identified. We gained insight from participants and physical therapists at the conclusion of the trial that have guided this discussion.

Participant implementation obstacles included: (1) Education provided by the VR-PNE headset was difficult to hear during clinic operation and auxiliary headphones were not available. (2) The extra time required for VR PNE at the end of the session was inconvenient to participants’ personal schedule. (3) Some analogies used during PNE sessions were difficult to understand or were described as patronizing.

Therapist implementation obstacles included two major themes: therapist work schedule and technological issues. Schedule issues included (1) Set up time, including the education on technology, added time stress to an already busy work schedule. (2) Balancing multiple patients made remembering who was enrolled in the study more difficult. (3) Participants arriving late would often not receive VR-PNE sessions due to other elements of care being prioritized above VR-PNE.

Technology-related issues included: (1) Wi-Fi connectivity issues that disrupted study procedures and workflow. (2) Session data would not launch at times for unknown reasons. (3) Therapist unfamiliarity with product troubleshooting, e.g. recentering visual field with remote when participant removed headset and changed position. (4) Lack of consistent charging procedures resulted in inability to launch sessions at the end of day or the following morning.

### Research

Several important findings regarding primary aims in this feasibility trial can be used to improve an expanded implementation of this trial. (1) Our overall recruitment rate indicated that a recruitment time of 3 years would have been required to conduct a fully-powered effectiveness trial. Future design should include more locations with higher volumes with highly engaged clinical staff. (2) Nine different outcome tools were used and may have created survey fatigue and contributed to the relatively low proportion of completed assessments. (3) The screening process did require considerable effort and support from the research team. Clinical sites completing screening and consent processes could improve allocation efforts by eliminating one hand-off point. Including additional educational sessions to review procedures for new employees may also assist with overall recruitment. (4) Attrition rates were high and could be improved with refined research methodology. Better motivational strategies, including motivational interviewing techniques during recruitment and providing financial compensation for providing study-related surveys could reduce attrition. (5) Patient compliance with VR-PNE sessions was better than anticipated with 73.7% completing six or more sessions. The optimal number of VR-PNE sessions is yet to be determined but may be more than six. Changes to the number of VR-PNE sessions should be considered sparingly to balance the participant burden.

Secondary aims also revealed some useful themes. (1) Narrowing the scope to more meaningful outcomes based on directionality of movement could improve follow up reporting (e.g. ODI, etc.). (2) Though group comparisons were only described, the data suggests there may be added value of VR-PNE. VR-PNE satisfaction and visit averages were greater than the PT as usual group indicating that the intervention was well tolerated.

## Strengths

This study had several strengths that should be highlighted. (1) Multicenter design enhances the generalizability and diversity in the study population. (2) Pragmatic design demonstrates practices that would closely mimic real-world application. (3) Randomized and controlled methodology minimizes bias and improves reliability of the findings. Lastly, this study demonstrated that outpatient providers, researchers and product developers can collaborate without grant funding to enhance treatment of adults with CLBP.

## Limitations

During this trial several limitations were noted: (1) Capturing baseline data after consent was difficult because participants did not complete their baseline survey prior to the second PT visit and would become ineligible. A longer acceptable timeframe for enrollment or direct clinic enrollment could be considered. (2) Clinician attrition made it difficult to keep stable rolling recruitment. Many clinicians’ job status changed during the trial. Including more clinics with higher CLBP patient volume should minimize the impact of clinician attrition. (3) Follow-up outcomes were difficult to capture ad-hoc. A common reason for not completing was the loss of the emailed follow-up link. Participants often verbally reported that they would complete it but did not. After three phone calls, patients could not be contacted and would become lost to follow-up. Providing links *via* text messages may improve compliance for future efforts. (4) Technical or user difficulties with the device. At times clinicians reported struggling with timing for set up or trouble with patients losing orientation of the VR-PNE field. Furthermore, VR-PNE is not a common tool present in most clinics, nor is it commonly used in entry level education potentially impacting the feasibility of its use by clinicians. (5) Competing interests for clinicians. Clinicians were rolling out new company policy and procedures and reported that scheduling conflicts influenced ability to deliver VR-PNE sessions each visit. (6) Duration of VR-PNE intervention. The program was designed to improve understanding of pain through 12 VR-PNE sessions; however, our design considered the use of an investigational dose of six sessions as acceptable. Future studies should consider utilizing more sessions. (7) Prior use of VR-PNE, sense of presence, nausea, dizziness, feeling of goggles, safety etc. was not formally assessed in participant questionnaires, but no adverse events were reported to clinicians. Future iterations of this trial should formally investigate any adverse events. (8) Lack of similar technology used in control group. VR-PNE is a newer, unique tool used in the clinical setting and could influence one’s outcomes. This study’s control group does not include use of similar, “cool” technology like VR. Future studies might want to use an immersive VR technology in their control group, but with some other experience than PNE. (9) Unequal randomization between groups. The randomization scheme was individualized by clinical site. Due to low recruitment numbers, our randomization was unequal. For a larger trial, we will use a study-wide randomization scheme versus one for each clinical site. (10) Finally, clinician engagement was likely limited due to uncertainty about the recruitment process. The study team received many questions about basic research processes throughout the recruitment timeframe. The additional time taken to reach out to the research team likely made several patients ineligible, and the likelihood that even more clinicians were not proficient in the recruitment process is high. This will be mitigated in future effort by providing a manual for clinicians and providing easy-to-find graphics for clinic bulletin boards.

## Conclusion

The results of the trial suggest that VR-PNE may be acceptable and feasible for patients with CLBP but fundamental changes to the study design are necessary prior to scaling up a follow up trial. When participants chose to continue PT, they continued to use VR-PNE education, but the rate of PT attendance could be improved. Additionally, the percentage completion of study assessments was proportionally low and improving follow up completion should be emphasized to reduce missing data. Reducing survey burden by decreasing the number of outcome tools used and sending participants text reminders for sessions and surveys may improve PT attendance and assessment completion rates. The VR-PNE education does not need to change but could be modified to a home component in a future trial to improve flexibility of delivery and decrease external distractions when bundled with PT sessions. To achieve the required sample size to determine between group differences, a longer recruitment duration and improved research strategies to improve follow-up rates are required. Due to the nature of feasibility trials, the secondary aims of this study should be considered as hypothesis generating.

## Data Availability

The data that support the findings of this study are available from the corresponding author, [RM], upon reasonable request.

## Appendix A1. Detail of VR-PNE curriculum modules

**Table:**

Lesson	Lesson learning objective	Module name	Module learning objective	Image	Duration
1	Patients are introduced to the concept of pain neuroscience and the tools used to manage pain.	Introduction to Pain Neuroscience Education	Patients listen to Dr. Louw introduce the Pain Neuroscience Education program.		29 min
		Cathy’s Patient Testimonial	Patients listen to Cathy’s story about her experience with persistent pain, Pain Neuroscience Education, and how it’s impacted her life.		
		Top Problems Assessment	Patients are assessed on their top problems associated with their pain.		
		Deep Breathing Exercise	Patients practice deep breathing exercises to help with their emotional regulation.		
		Introduction to Mindfulness	Patients experience their first guided mindfulness session, in the immersive environment. The session focuses on mindful relaxation.		
2	Patients learn more about the nervous system’s role in pain, *via* both 2-dimensional educational videos viewed in the VR environment, and 3-dimensional, immersive, educational experiences.	Motivational Interview	Patients will be asked questions related around their pain journey.		22 min
		Introduction to Nervous System	Patients will hear from an expert about how their nervous system works and the impacts on pain.		
		Cognitive Behavioral Therapy: Airplane Experience	Patients experience a cognitive behavioral therapy lesson using an airplane metaphor.		
		Mindfulness Practice 2	Patients experience their second mindfulness session, focused on mindful breathing.		
3	Patients further their knowledge of the correlation between pain and the nervous system through more educational metaphors and mindful practice.	Sensitive Alarm System	Patients learn about their “Sensitive Alarm System”.		18 min
		Mindfulness Practice 3	Patients experience their third Mindfulness session, consisting in a body scan mindfulness exercise.		
4	Patients expand their knowledge of the sensitivity of pain and how to better manage it.	How to Know if Your Nervous System is Too Sensitive	Patients learn how to know if their nervous system is too sensitive.		15 min
		How to Turn Down Your Nervous System Sensitivity	Patients learn how to turn down their sensitive nervous system through knowledge and exercise.		
		Mindfulness Practice 4	Patients experience their fourth Mindfulness session, consisting in an awareness of breath meditation.		
5	Patients learn additional factors and lifestyle changes that impact pain sensitivity.	Turn Down Your Nervous System Sensitivity 2	Patients learn how to turn down their sensitive nervous system through medication and sleep, in addition to knowledge, emotion regulation, and exercise.		22 min
		Mindfulness Practice 5	Patients experience their fifth Mindfulness session, consisting in an exercise focused on thoughts and emotions.		
6–12	Patients learn additional techniques and metaphors for pain management, tailored to the type of pain they’ve indicated during Lesson One assessments.	Brain Meeting	Dr. Louw teaches a lesson on brain science		22 min
		Lion & Stress	Introduction to threat mechanisms & stress chemicals		
		Nerve Sensors	Explanation of nerve sensors & how they are triggered to cause pain		
		Nosy Neighbors	Education on how pain spreads throughout the body		
		Custom Patient Testimonial	Patients view one of the four narratives based on their top problems assessment in lesson 1: Wesley’s Testimonial about how her injury caused issues with her focus and concentration, and how the program helped regulate her emotions and thoughts. Jennean’s Testimonial about the impact persistent pain had on her energy levels, and how she used the methods taught in the program to manage her pain and help her sleep again. Linda’s Testimonial about persistent plain, the pain flare ups her experience during times of stress and illness, and how she uses the program to manage her pain levels. TK's experience with widespread pain due to a series of serious injuries, and her journey to live an active life again by understanding her pain using the program.		
		Mindfulness Practices 6 to 12	Patients experience the following Mindfulness sessions: Letting go of resis-tance Riding the waves Shifting thoughts and feelings Riverbank meditation Awareness of awareness Putting it all together Self-compassion meditation		
